# A Gated Attention-Based Multiple Instance Learning and Test-Time Augmentation Approach for Diagnosing Active Sacroiliitis in Sacroiliac Joint MRI Scans

**DOI:** 10.3390/jcm15062101

**Published:** 2026-03-10

**Authors:** Zeynep Keskin, Onur İnan, Ömer Özberk, Reyhan Bilici, Sema Servi, Selma Özlem Çelikdelen, Mehmet Yıldırım

**Affiliations:** 1 Department of Radiology, Konya City Hospital, Konya 42020, Turkey; omerozberk@gmail.com; 2 Department of Computer Engineering, Faculty of Technology, Selçuk University, Konya 42250, Turkey; oinan@selcuk.edu.tr (O.İ.); semaservi@selcuk.edu.tr (S.S.); 3 Department of Rheumatology, Faculty of Medicine, Necmettin Erbakan University, Konya 42090, Turkey; drreyhanbilici@gmail.com; 4 Department of Internal Medicine, Konya City Hospital, Konya 42020, Turkey; drozlemkoc@hotmail.com (S.Ö.Ç.); mdmehmetyildirim476@gmail.com (M.Y.)

**Keywords:** sacroiliitis, axial spondyloarthritis, magnetic resonance imaging, gated attention, multiple instance learning

## Abstract

**Background and Objective:** Axial spondyloarthritis (axSpA) is a group of chronic inflammatory diseases that primarily affect the sacroiliac joints. Early diagnosis is crucial for preventing irreversible structural damage. Magnetic Resonance Imaging (MRI) is the gold standard for detecting early inflammatory changes such as sacroiliitis. However, conventional MRI interpretation is inherently subjective and susceptible to both intra- and inter-observer variability. Therefore, artificial intelligence (AI)-driven diagnostic solutions are increasingly being explored. Among them, the Gated Attention Multiple Instance Learning (MIL) framework holds strong potential in modeling heterogeneous inflammatory distributions, thanks to its slice-level attention mechanism. This study aims to evaluate the diagnostic performance of a deep learning model based on Gated Attention MIL for automated sacroiliitis detection. Furthermore, its results are compared with a baseline deep learning architecture (standard ResNet-18), and its consistency with radiologist annotations is analyzed. **Materials and Methods:** The dataset included 554 subjects, comprising 276 patients diagnosed with axSpA and 278 healthy controls. All MRI data were derived from axial T2-weighted fat-suppressed (T2_TSE_TRA_FS) sequences. Patient-wise data splitting was employed to construct training, validation, and independent test sets. The proposed model architecture integrates ResNet-18-based feature extraction, a gated attention mechanism for instance-level weighting, and bag-level classification. Additionally, Test-Time Augmentation (TTA) was implemented to enhance robustness during inference. **Results:** On the independent test set, the model achieved an accuracy of 85.88%, sensitivity of 92.86%, specificity of 79.07%, and an F1-score of 86.67%. Attention heatmaps generated by the MIL module showed strong spatial overlap with bone marrow edema regions annotated by expert radiologists. Implementation of TTA led to an approximate 10% improvement in overall classification accuracy. **Conclusions:** The Gated Attention MIL framework demonstrated high diagnostic performance for sacroiliitis detection, indicating its value as a reliable decision support tool for early axSpA diagnosis. Validation on larger, multi-center datasets is warranted to ensure generalizability and to support clinical integration in routine radiology workflows.

## 1. Introduction

Axial spondyloarthritis (axSpA) is a group of chronic inflammatory rheumatic diseases primarily affecting the spine and sacroiliac joints, typically beginning in early adulthood and potentially leading to progressive structural damage and functional impairment [1]. Early diagnosis is of critical importance to alter the natural course of the disease and improve long-term functional outcomes. However, conventional radiography often appears normal in the early stages of axSpA, resulting in diagnostic delays [2]. 

According to the European League Against Rheumatism (EULAR), the definition of sacroiliitis on Magnetic Resonance Imaging (MRI) in patients with SpA is based on the qualitative, i.e., visually appreciable, presence of subchondral bone marrow edema (BME) as an indicator of active inflammation. Within the framework of the Assessment of SpondyloArthritis International Society (ASAS) classification criteria, MRI’s ability to detect inflammation in the sacroiliac joints is highlighted as crucial for diagnosis [3]. MRI can reveal bone marrow edema and signs of active inflammation much earlier than radiographic imaging. However, interpreting MRI scans is time-consuming and inherently subjective. Differences in interpretation between expert radiologists and rheumatologists may arise during image assessment [4]. These discrepancies can introduce uncertainty in the diagnostic and therapeutic decision-making process, potentially compromising diagnostic accuracy.

Despite efforts toward standardization and the development of guidelines such as those by ASAS [3], qualitative and semi-quantitative (i.e., measurable but not fully numerical) assessments remain largely dependent on the observer’s interpretation. As a result, intra- and inter-observer variability persists in the image analysis process, continuing to pose diagnostic challenges [4]. Although ASAS imaging criteria aim to improve standardization, in early-stage or borderline cases, MRI assessments still rely heavily on observer experience. Consequently, objectivity and reproducibility remain limited [4,5].

In recent years, artificial intelligence (AI) and deep learning techniques have emerged as promising tools to overcome these limitations in medical imaging. Convolutional Neural Networks (CNNs) possess the capability to analyze textual and morphological features of images at a resolution beyond human visual perception [6]. However, the heterogeneous distribution of lesions in sacroiliitis reduces the efficiency of classical CNN models that process all slices uniformly. Therefore, the Multiple Instance Learning (MIL) framework is particularly well-suited, as it enables the model to treat each patient as a “bag” of instances (i.e., slices) and learn the importance of each slice dynamically. Gated Attention MIL, in particular, generates attention weights across slices, allowing the model to emphasize inflamed regions [7]. AI algorithms can thus accelerate the automated analysis of MRI data, grading of disease activity, and detection of inflammatory changes in the sacroiliac joints.

Recent advances in deep learning for medical image analysis have increasingly focused on improving model robustness and generalization under heterogeneous data distributions and multi-center settings. In particular, personalized federated learning (FL) frameworks have been proposed to address inter-institutional variability while preserving data privacy. For example, PHH-FL introduces a perceptual hashing–based similarity mechanism combined with a hypernetwork architecture to dynamically generate personalized parameters for heterogeneous medical imaging tasks, effectively balancing personalization and cross-client generalization [8]. Such approaches highlight the growing importance of adaptive and distribution-aware architectures in collaborative medical imaging environments.

In parallel, knowledge distillation (KD) and teacher–student learning paradigms have emerged as influential strategies in modern medical image analysis. A recent comprehensive overview by [9]. systematically analyzes KD-based methods across classification, segmentation, detection, reconstruction, and report generation tasks, emphasizing that KD extends beyond model compression to roles such as semi-supervised learning, weak supervision, class balancing, and modality compensation. These findings underline the increasing relevance of structured knowledge transfer mechanisms in developing robust and computationally efficient medical imaging models.

These recent developments demonstrate that contemporary medical image analysis research increasingly emphasizes robustness, generalization, and adaptive learning strategies. However, for slice-based MRI classification tasks such as sacroiliitis detection, modeling intra-patient heterogeneity and identifying diagnostically relevant slices remain critical challenges. In this context, attention-guided MIL frameworks offer a practical and interpretable solution for capturing localized inflammatory patterns within volumetric MRI data.

This study aims to evaluate the diagnostic performance of a Gated Attention MIL-based deep learning model combined with Test-Time Augmentation (TTA) for detecting active inflammatory sacroiliitis on sacroiliac joint MRI scans.

## 2. Materials and Methods

This study was designed as a retrospective, single-center, observational investigation. The study was conducted in accordance with the principles of the Declaration of Helsinki and received approval from the local ethics committee (Decision No: 2024/001, date: 19 November 2024). Although the MRI examinations were performed between November 2022 and October 2024 as part of routine clinical practice, no data processing or analysis was conducted prior to obtaining ethics committee approval. Due to the retrospective nature of the study, the requirement for obtaining informed consent from patients was waived by the ethics committee. All imaging data and clinical information included in the study were obtained from the hospital’s electronic medical record system, and all data were fully anonymized prior to analysis.

### 2.1. Study Population and Inclusion Criteria

A total of 554 individuals aged over 18 who presented to our hospital’s Rheumatology Outpatient Clinic between November 2022 and October 2024 were included in this study. Of these, 276 were patients diagnosed with axSpA, while 278 were clinically and radiologically confirmed healthy controls. The diagnosis of axSpA was established by consensus among one rheumatologist and two radiologists based on the ASAS 2009 classification criteria [3].

### 2.2. Study Population and Selection Criteria

This study retrospectively included cases that met specific inclusion and exclusion criteria. The inclusion criteria were as follows: age 18 years or older, availability of sacroiliac joint MRI, full accessibility of clinical records, and the presence or absence of acute inflammatory sacroiliitis clearly reported radiologically. For both the active sacroiliitis group and the healthy control group, all MRI examinations were independently reviewed by two experienced radiologists and one rheumatology specialist, and the final classification was established through multidisciplinary consensus among the three experts.

Exclusion criteria included significant artifacts or low-resolution issues in MRI, history of any surgical intervention on the sacroiliac joint, pathological conditions requiring differential diagnosis such as fractures, and the use of MRI data obtained from sequences outside the standard imaging protocol. In accordance with these criteria, only cases with diagnostically valuable, appropriate, and standardized imaging were included in the study.

### 2.3. Imaging Protocol

**Figure 1:**
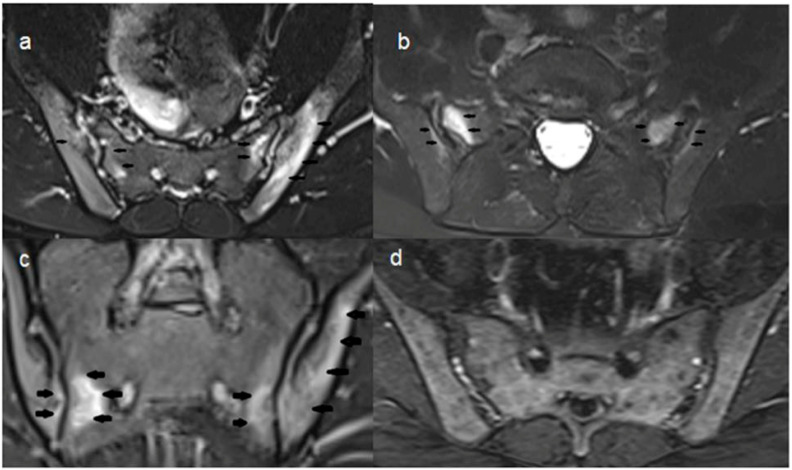
Examples of sacroiliac joint imaging. In individuals with active sacroiliitis, axial T2 TSE FS sequences of (**a**) a 19-year-old and (**b**) a 49-year-old male subject, as well as (**c**) a coronal T2 TSE FS sequence of a 19-year-old male patient, demonstrate marked hyperintense signal increases in the bilateral sacroiliac joints—particularly in the subchondral regions of the sacral and iliac bones—findings consistent with active sacroiliitis (indicated by arrows). (**d**) In the coronal T2 TSE FS sequence of a 40-year-old healthy female subject, no pathological hyperintensity is observed.

All MRI scans were performed using a 1.5 Tesla Magnetom Vision scanner (Siemens Medical Systems, Erlangen, Germany) located at the same center. For sacroiliac joint evaluation, the T2-TSE Transverse Fat-Suppressed (t2_tse_tra_FS) sequence was selected in accordance with the protocol recommended by ASAS. This sequence is recognized in the literature for its high sensitivity in BME (Figure 1) [3].

Imaging parameters were as follows: TR/TE time of 2220/66 ms, slice thickness of 3.3 mm, matrix size of 110 × 256, and field of view (FOV) of 250 mm. For each individual, an average of 25 to 60 transverse (axial) slices was obtained. All DICOM data were converted to 8-bit grayscale PNG format using a Python-based converter, resized to 224 × 224 pixels, normalized to a 0–255 pixel value range, and subjected to histogram equalization for contrast standardization.

### 2.4. Dataset and Preprocessing

The dataset consisted of 554 individuals (patients and healthy controls) who underwent MRI for suspected sacroiliitis. Data were extracted from ISO files in the hospital’s PACS system using automated scripts, selecting only the t2_tse_tra_FS sequence. Images were organized in individual folders for each subject, creating a “bag” structure. Each bag contained a minimum of 25 slices.

To prevent data leakage, the dataset was split on a patient basis into training, validation, and test subsets. The training set included 193 patients and 194 healthy controls (387 subjects total) with 11,677 slices. The validation set included 41 patients and 41 healthy controls (82 subjects total) with 2473 slices. The test set comprised 42 patients and 43 healthy controls (85 subjects total) with 2549 slices. Across all datasets, a total of 16,699 images from 276 patients and 278 healthy controls were used. There was no overlap of individuals between training, validation, and test sets. During training, data augmentation techniques such as random horizontal and vertical flipping, contrast and brightness variations were applied to prevent overfitting. All images were normalized according to ImageNet statistics.

### 2.5. Deep Learning Architecture: Gated Attention MIL

The proposed Gated Attention MIL model’s performance was evaluated against a vanilla ResNet-18 model, which served as the primary baseline to isolate the specific impact of the gated attention mechanism.

Given that sacroiliitis typically exhibits focal or patchy involvement without affecting the entire tissue uniformly, a MIL-based architecture was employed instead of conventional supervised learning [10]. In this approach, each patient is considered a “bag” and their MRI slices are treated as “instances” within that bag.

**Figure 2:**
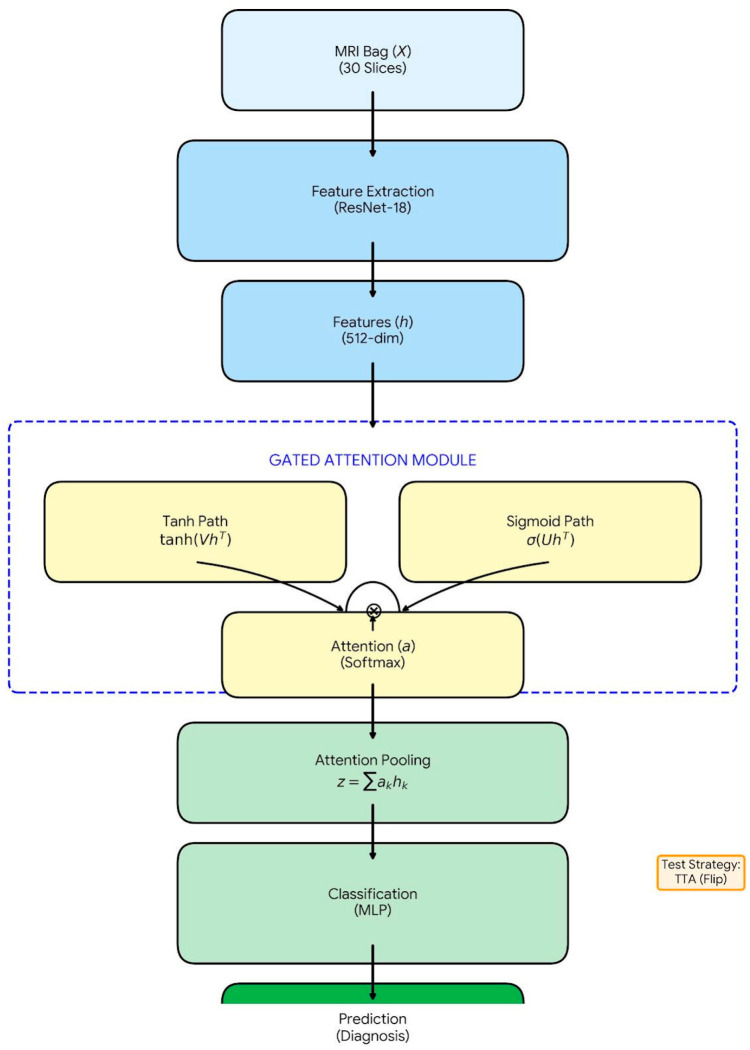
Architectural flow diagram of the proposed Gated Attention MIL framework.

The proposed diagnostic framework utilizes a Gated Attention Multiple Instance Learning (MIL) architecture, as illustrated in Figure 2. The model architecture consists of three main stages: (1) Feature Extraction, (2) Gated Attention Mechanism, and (3) Bag-Level Classification.

1. Instance-Level Feature Extraction: Each patient P_i__, is represented as a bag containing K MRI slices. A deep Convolutional Neural Network (CNN) was employed to transform the inflammatory tissue characteristics (such as intensity variations and structural distortions) present in the image $X_{i}=\{x_{i1},x_{i2},\ldots ,x_{iK}\}$ to a low-dimensional feature vector ($h_{ik}$) [11]. The ResNet-18 model, pre-trained on ImageNet, was employed as the backbone architecture [12]. The fully connected layers of the model were removed, and a feature vector of dimension (d = 512) was extracted for each slice:
  $$h_{ik}=f_{ResNet}\left(x_{ik}\right),\;\;\;h_{ik}\in \;R^{512}$$
2. Gated Attention Mechanism: The most critical step in detecting active sacroiliitis is identifying which slices in the MRI series show signs of inflammation. A Gated Attention Mechanism was employed to prevent healthy slices from negatively influencing the model’s decision.

This mechanism calculates a learnable attention score ($a_{k}$) for each slice k, indicating how informative that slice is for the patient’s inflammation status. It combines hyperbolic tangent (tanh) and sigmoid (σ) activation functions:

$$a_{k}=\frac{exp\;\{w^{T}\left(\tanh\left(Vh\frac{T}{k}\right)⊙sigm\left(Uh\frac{T}{k}\right)\right)\}}{\sum_{j=1}^{K}exp\;\{w^{T}\left(\tanh\left(Vh_{j}^{T}\right)⊙sigm\left(Uh_{j}^{T}\right)\right)\}}$$

Here: w, V, U are trainable weight matrices.

⊙ represents element-wise multiplication [12].

This allows the model to assign low weights (near zero) to non-informative slices and high weights to potentially inflamed slices, thereby mitigating mislabeling issues [13].

3. Bag Representation and Classification: A single patient-level feature vector (*z_i_*) is obtained by aggregating all instance feature vectors weighted by their attention scores ($a_{k}$)

$$z_{i}=\sum_{k=1}^{K}a_{k}h_{ik}$$

This *z_i_* vector contains the most discriminative inflammation-related features for that patient. Finally, this vector is passed through a classifier layer to compute the probability of being “Inflamed” or “Healthy” [11,12,13,14,15].

### 2.6. Test-Time Augmentation (TTA)

To enhance the model’s robustness against noise and acquisition variability, TTA was applied during inference. Each patient’s slices were presented to the model in both their original and horizontally flipped forms, with final predictions obtained by averaging the outputs [16,17].

### 2.7. Training and Implementation Details 

The experiments were performed in the Google Colab cloud environment (Google LLC, Mountain View, CA, USA) using Python 3.12.12 (Python Software Foundation, Wilmington, DE, USA). The deep learning model was implemented using a pre-trained ResNet-18 architecture via PyTorch (v2.10.0) and Torchvision (v0.25.0) libraries (Meta Platforms, Inc., Menlo Park, CA, USA). Image processing operations were conducted using Pillow (v11.3.0) (Python Software Foundation, Wilmington, DE, USA), while numerical computations were performed with NumPy (v2.0.2). Model evaluation metrics, including accuracy, F1-score, and confusion matrix calculations, were computed using Scikit-learn (v1.6.1). The detailed hyperparameter configurations and implementation settings used in this study are summarized in Table 1 to enhance methodological transparency and reproducibility. The ImageNet-pretrained ResNet-18 backbone was fully fine-tuned on our dataset, with no layers frozen during training, to optimize feature representation for sacroiliac MRI analysis.

The gated attention module was configured with an internal attention dimension of D=128, projecting 512-dimensional instance-level features (L=512) to a single attention weight (K=1) per slice.

The network was optimized using the Adam optimizer with a learning rate of $1\times 10^{-4}$, weight decay of $1\times 10^{-3}$, and a batch size of four patient-level bags over 20 epochs. Model selection was performed based on the highest validation F1-score. Patient-level classification was trained using Cross-Entropy loss.

As the dataset exhibited a near-balanced class distribution, no explicit class balancing strategy (e.g., oversampling or weighted loss) was applied. During inference, Test-Time Augmentation (TTA) using deterministic horizontal flipping was employed to improve prediction stability.

### 2.8. Statistical Analysis

To justify the adequacy of the independent test cohort, an AUC-based statistical power estimation was performed. Using the observed AUC of 0.9468 and the class distribution of 42 positive and 43 negative cases, the estimated standard error of the AUC was approximately 0.027. This yields a Z statistic of approximately 16 when tested against a null hypothesis AUC of 0.5. At a significance level of α = 0.05, this corresponds to statistical power greater than 0.99. These results support that the independent test sample size is sufficient to reliably estimate model discrimination performance.

### 2.9. Findings

In the cohort of 554 individuals (mean age: 37.9 years; range: 18–76), the Gated Attention MIL-based deep learning model demonstrated high diagnostic accuracy in detecting active inflammatory sacroiliitis on sacroiliac joint MRI. The MRI and structural imaging findings, clinical and laboratory characteristics, and the coexistence of active osteitis with rheumatic diseases in the study population are summarized in Table 2, Table 3 and Table 4. A total of 16,699 MRI slices obtained from these 554 unique individuals were divided into training (*n* = 11,677), validation (*n* = 2473), and test (*n* = 2549) sets on a per-patient basis. This patient-level splitting strategy completely eliminated the risk of data leakage and enabled an objective and reliable assessment of the model’s generalization performance under real-world clinical conditions.

This table summarizes the distribution of rheumatic diseases in 276 patients with active osteitis, including adjusted patient counts, sex distribution, and mean age by sex.

**Figure 3:**
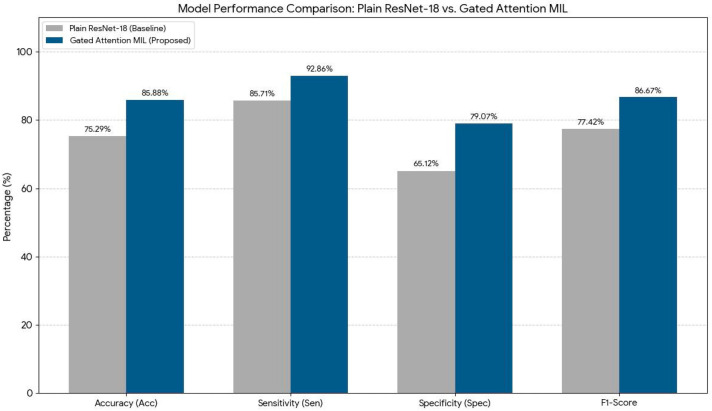
Comparison of Model Performance Metrics Between Baseline ResNet-18 and Proposed Gated Attention MIL.

**Figure 4:**
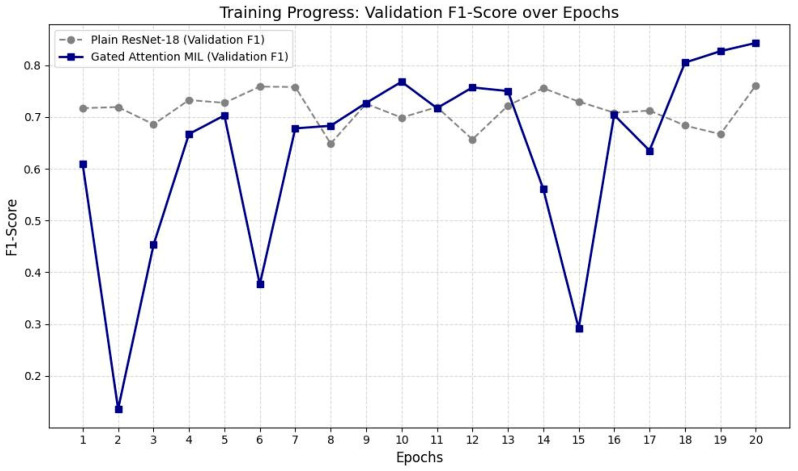
Validation F1-Score Across Training Epochs for ResNet-18 and Gated Attention MIL Models.

The model’s baseline accuracy prior to the application of TTA was approximately 75.29%. Following the implementation of TTA, this rate increased to 85.88% (Table 5). This improvement indicates that the model became more resilient to variables such as image quality, anatomical variations, and borderline inflammatory signal changes. These findings suggest that TTA significantly enhances the model’s generalization capacity and strengthens diagnostic consistency despite clinical variability (Figure 3 and Figure 4).

Bar chart comparing baseline ResNet-18 and Gated Attention MIL. The proposed model outperforms across all metrics: accuracy, sensitivity, specificity, and F1-score.

This line graph presents the validation F1-scores of the baseline ResNet-18 and the proposed Gated Attention MIL model over 20 training epochs. While ResNet-18 reaches an early performance plateau, the Gated Attention MIL model demonstrates a dynamic learning pattern with eventual superior performance, reflecting its capacity to focus on informative image slices over time.

### 2.10. Analysis of F1-Score Training Logs and Model Performance

Upon examining the F1-score logs from the training process, a characteristic difference in learning behavior was observed between the baseline ResNet-18 architecture and the proposed Gated Attention MIL model. The baseline ResNet-18 model reached performance saturation (plateau) as early as the 5th epoch and exhibited unstable fluctuations in the range of 64% to 76% throughout the training. These fluctuations in the standard CNN architecture are attributed to its equal-weighted, patient-independent processing of each MR slice, which results in clinically non-informative slices introducing noise into the model and limiting its generalization capacity.

In contrast, the Gated Attention MIL model demonstrated lower initial performance and experienced occasional abrupt score drops in the early training phases. These temporary instabilities are due to the model’s initial inability to optimally calibrate the “gated attention” coefficients, occasionally assigning high attention weights to noisy slices lacking inflammatory signals. In the hierarchy of multiple instance learning, even a single misweighted slice can significantly impact the overall patient-level (bag-level) decision, manifesting as short-term fluctuations in the training curve.

However, from the 10th epoch onward, the model refined its weighting mechanism and entered a more stable upward trend. Particularly after the 18th epoch, the model showed accelerated improvement, achieving a validation F1-score of 84.3%. This demonstrates that the gated attention mechanism progressively learned to effectively isolate slices containing inflammation-critical regions essential for the diagnosis of sacroiliitis. In contrast to the stagnant performance of the baseline model, the dynamic and selective learning process of the MIL model yielded a much deeper and higher-resolution feature representation, particularly advantageous for images with heterogeneous pathological distributions such as the sacroiliac joint.

Results from the independent test set confirmed that the proposed MIL approach significantly outperforms standard deep learning methods in diagnostic accuracy. The accuracy rate of the baseline ResNet-18 model (75.29%) improved to 85.88% with the Gated Attention MIL architecture, representing an absolute increase of approximately 10.6%. The most notable improvement was observed in specificity, which increased by 13.95% to reach 79.07%. This enhancement highlights the model’s ability to filter out non-inflammatory slices and healthy joint structures by assigning them lower weights, thus reducing false positives (Table 6). Furthermore, the sensitivity rate reaching as high as 92.86% underscores the model’s success in detecting early-stage sacroiliitis cases and reinforces its potential as a reliable screening tool in clinical decision support systems. Based on these findings, it can be concluded that attention-based multiple instance learning offers substantially superior generalization and diagnostic capabilities compared to standard image classification models, particularly in anatomically complex regions such as the sacroiliac joint where pathology is heterogeneously distributed.

**Figure 5:**
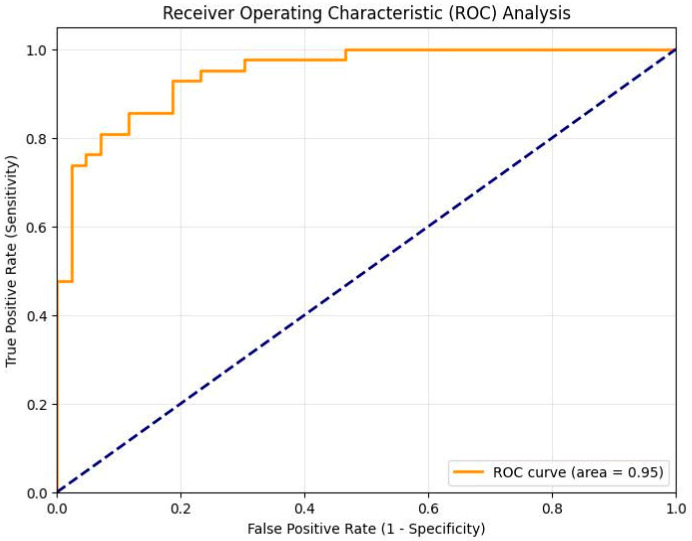
Receiver Operating Characteristic (ROC) Curve of the Proposed Gated Attention MIL Model on the Independent Test Set.

The overall diagnostic performance of the proposed Gated Attention MIL framework was evaluated using multiple metrics on the independent test set. The model achieved an outstanding AUC score of 0.9468, as illustrated in the Receiver Operating Characteristic (ROC) analysis (Figure 5).

This high AUC value, combined with a sensitivity of 92.86% and an accuracy of 85.88%, confirms the model’s robust capability to discriminate between active sacroiliitis and healthy controls. The detailed classification results, including the confusion matrix, are presented in Table 5.

The ROC curve demonstrates the discrimination performance of the proposed model for sacroiliitis detection. The area under the curve (AUC) was 0.9468, indicating excellent classification capability. The model achieved a sensitivity of 92.86% and specificity of 79.07% at the selected operating threshold. The confusion matrix results (TP = 39, TN = 34, FP = 9, FN = 3) further confirm strong diagnostic performance with a low false-negative rate.

### 2.11. Model Interpretability Analysis

**Figure 6:**
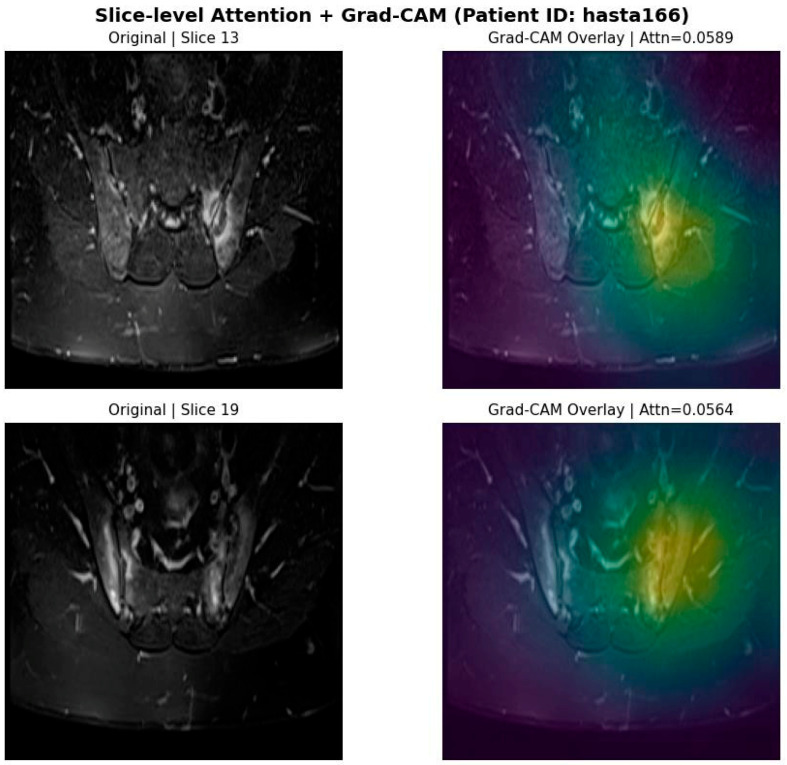
Spatial interpretability using Grad-CAM (Patient ID: hasta166).

To demonstrate the clinical interpretability of the proposed architecture, we analyzed the attention mechanisms at both the bag (slice) and instance (pixel) levels. Figure 6 illustrates the slice-level attention weights generated by the MIL pooling module for a representative patient. The model assigns the highest scores to slices capturing the sacroiliac joint region, while suppressing uninformative slices (e.g., lower lumbar or predominantly muscular regions) with near-zero weights.

**Figure 7:**
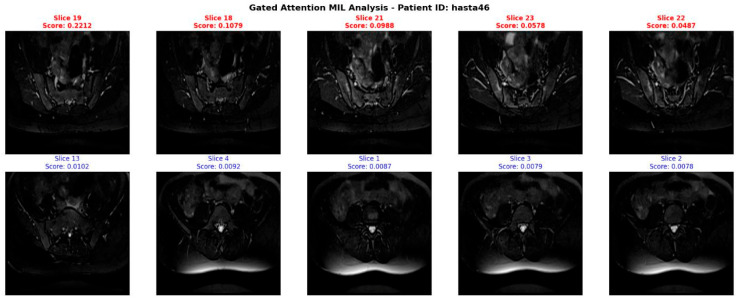
Slice-level attention analysis (Patient ID: hasta46).

To further investigate spatial localization, Gradient-weighted Class Activation Mapping (Grad-CAM) was applied to the top-attended slices. As shown in Figure 7, the spatial heatmaps highlight regions corresponding to bone marrow edema within the sacroiliac joints. Visual inspection by an expert radiologist confirmed qualitative agreement between the Grad-CAM activation regions and clinically identified inflammatory areas.

The left column shows the original MRI slices receiving high attention weights. The right column presents the corresponding Grad-CAM overlays, demonstrating that the model’s spatial focus is concentrated in regions consistent with bone marrow edema identified by expert radiologists.

The top row displays the five slices with the highest attention weights a_k_, corresponding to clinically relevant sacroiliac joint regions. The bottom row shows the five lowest-weighted slices, effectively suppressed by the model due to limited pathological information.

## 3. Discussion

This study quantitatively demonstrated the superiority of the Gated Attention MIL architecture developed for the diagnosis of active sacroiliitis in sacroiliac joint MRIs, compared to standard deep learning approaches (i.e., plain ResNet-18). The results show that our model achieved an accuracy of 85.88% and a high sensitivity of 92.86% on the independent test set. These performance values significantly surpass the outcomes reported for classical CNN architectures in the literature.

The most striking finding of our study is the Gated MIL model’s improvement of classification accuracy by 10.59% and specificity by 13.95% compared to the plain ResNet-18. The baseline ResNet-18 model, treating all MR slices with equal weight, processes signals from healthy, non-inflammatory slices as “noise,” leading to a notably low specificity of 65.12%. In contrast, the Gated Attention mechanism filters out this noise by focusing only on clinically meaningful slices (e.g., those BME) in sacroiliitis cases with heterogeneous lesion distributions.

When compared with similar studies in the literature, for example, the classical CNN model developed by Bordner et al. (2023) reported a sensitivity of 56%, whereas the use of MIL in our study increased this value to 92.86% [7]. This demonstrates that “bag-level” learning, as opposed to “instance-level” learning, provides more reliable results in clinical decision support systems for focal pathologies like sacroiliitis.

The high sensitivity of the model helps minimize the risk of “missed cases” (false negatives), which is critical in early axSpA diagnosis. Furthermore, the integration of TTA into the model boosted accuracy by approximately 10%, enhancing robustness against different acquisition parameters and anatomical variability.

Furthermore, while our model demonstrated high sensitivity in detecting active sacroiliitis, evaluating its performance on borderline or early-stage cases, as well as stratifying performance across different disease severities (e.g., mild vs. severe), remains a limitation of the current study. Our dataset primarily consisted of clinically confirmed active cases with binary labels (active vs. healthy), lacking granular severity grading (such as SPARCC scores). The fine-grained feature extraction and spatial focusing capabilities of our Gated Attention MIL architecture may offer potential advantages in capturing the subtle inflammatory signals characteristic of early-stage or mild disease. However, since identifying early-stage inflammation and quantifying disease severity are widely recognized challenges in recent AI literature, validating this potential on specifically curated cohorts with detailed clinical grading will be a primary focus of our future research.

In conclusion, despite being trained with weakly labeled data (i.e., patient-level labels only), the Gated Attention MIL model provides high diagnostic reliability and time efficiency, unlike methods requiring complex segmentation or manual ROI annotation. This study supports the potential of the proposed AI solution to serve as a standardized assistive tool in clinical practice by reducing observer-dependent variability in radiological interpretation.

The proposed Gated Attention MIL deep learning model demonstrates high diagnostic accuracy in detecting active inflammatory sacroiliitis in sacroiliac joint MRIs. In the independent test set, which included 85 previously unseen patients, the model achieved 85.88% accuracy, 92.86% sensitivity, and 79.07% specificity. These results are significantly better than comparable approaches in the literature and highlight the effectiveness of the MIL approach in modeling the heterogeneous and slice-distributed inflammatory patterns of sacroiliitis [5,6,7].

Previous studies have explored various AI techniques for sacroiliitis classification. For example, Faleiros et al. (2020) [6] used support vector machines (SVM) and random forest algorithms, reporting 75–82% accuracy. However, these models were limited by manual feature selection and observer dependency.

More recently, deep learning architectures have become more prevalent. Bordner et al. (2023) [7] developed a CNN model to automatically detect active sacroiliitis based on ASAS MRI criteria. While their model reported 56% sensitivity and 100% specificity in the external validation set, its AUC was only 0.76—indicating limited clinical utility due to low sensitivity. Classic CNNs evaluate all slices with equal importance, often missing critical slices in heterogeneous MRIs.

In a large-scale study by Bressem et al. (2022) [18], a deep learning framework was developed for detecting inflammatory and structural changes in the sacroiliac joints in axial spondyloarthritis (axSpA). With a training/validation dataset of 477 patients and an independent test set of 116, the model achieved 84% accuracy in the validation set and 75% in the independent test set for identifying active inflammation. For structural changes, the model achieved 85% and 79% accuracy, respectively, demonstrating reasonable generalization on new data [18].

In the study by Zhang et al. (2024) [19], the effectiveness of deep learning radiomics (DLR) combining multimodal MRI features with clinical data was evaluated. Their ensemble model, integrating ResNet50, ResNet101, and DenseNet121, achieved 82.5% accuracy. The final hybrid model combining the DLR signature with clinical factors achieved the highest diagnostic accuracy of 85.6% [19].

In another multi-center study by Bressem et al. (2022) [20], a deep learning model was developed to detect inflammatory and structural changes in sacroiliac joint MRIs in axSpA. Among 593 patients, the model achieved 75% accuracy for detecting inflammatory changes (sensitivity 88%, specificity 71%) and 79% for structural changes (sensitivity 85%, specificity 78%) on an external test set (*n* = 116). High AUC values (0.88–0.94 for inflammation and 0.89 for structural changes) support the model’s reliability in standardizing axSpA diagnosis and assisting radiologists [20].

In a retrospective cohort study by Liu et al. [21], a diagnostic model was developed using semi-supervised segmentation and radiomic analysis of sacroiliac MRIs for detecting sacroiliitis and BME. Radiomic features extracted from automated segmentations using the Unimatch framework were classified with SVM, logistic regression (LR), and Light GBM. Independent test results showed 81.2% accuracy for sacroiliitis and 74.2% for BME detection. This study demonstrated the potential of combining machine learning and radiomics to reduce reliance on expert annotation and enhance diagnostic workflows [21].

In another study by Nicolaes et al. (2025) [22], a deep learning algorithm was tested on a large external validation set of 731 patients drawn from two randomized controlled trials (RAPID-axSpA and C-OPTIMISE). Using expert assessments based on the 2009 ASAS definitions as a reference, the algorithm achieved 74% overall agreement (accuracy). On independent test data, the model achieved 70% sensitivity, 81% specificity, and 84% positive predictive value indicating acceptable performance for identifying inflammatory changes in large, heterogeneous datasets [22]. As summarized in Table 7, the Gated Attention MIL model achieved the highest independent test accuracy among the reviewed approaches.

This table summarizes machine learning, radiomics, and deep learning-based approaches used for the detection of sacroiliitis and axSpA from sacroiliac joint MRI, along with their performance on independent test datasets. The reviewed studies differ in dataset size, model architecture, and methodological design. Compared with previously reported methods, the proposed Gated Attention MIL approach achieves the highest independent test accuracy.

Although our primary experimental baseline was a slice-level ResNet-18 model trained on the same dataset, previously reported classical machine learning approaches (e.g., SVM and Random Forest models achieving 75–82% accuracy) provide contextual benchmarks in the literature. While direct dataset-level comparison across studies is inherently limited due to differences in cohort composition and evaluation protocols, our results demonstrate competitive and improved diagnostic performance relative to these reported approaches.

The primary advantage of the Gated Attention MIL approach used in this study lies in its ability to dynamically evaluate the importance of each image slice and assign higher weights only to the most informative ones. This mechanism is particularly meaningful given that sacroiliitis typically presents intensely in certain slices while remaining minimal in others. The architecture developed by Ilse et al. (2018) [10] enables learning from weakly labeled data, making it highly suitable for clinical applications. Similarly, our study achieved high classification performance using only patient-level labels under a comparable weak labeling scenario.

Inconsistencies in intra- and inter-observer evaluations are a significant challenge in diagnosing sacroiliitis [4]. In our study, MIL attention maps significantly overlapped with the BME regions identified by radiologists, suggesting the model not only performs classification but may also contribute to clinical decision-making. Given the risk of treatment delays from missed early inflammatory changes, the model’s high sensitivity (92.86%) is clinically vital. Its low false-negative rate further supports its ability to detect early inflammation.

Moreover, the model’s relatively low false positive rate (20.93%) suggests it could reduce unnecessary diagnostic procedures or treatments. The model’s specificity (79.07%) reflects its capacity to distinguish healthy individuals, minimizing misinterpretation of mechanical back pain or degenerative changes as inflammatory lesions.

The integration of TTA boosted accuracy by approximately 10%, enhancing the model’s robustness against image quality variations and acquisition inconsistencies. This finding aligns with other studies reporting improved generalization performance through TTA [24,25]. Given the variability in sacroiliac joint anatomy and imaging parameters, TTA’s contribution in this domain is clear.

Since MIL does not require slice-level annotations, its clinical applicability is high. Annotating individual slices is both time-consuming and expert-dependent. In our study, patient-level labeling sufficed due to MIL, significantly accelerating data preparation. Furthermore, the attention mechanism provided insight into which slices influenced predictions, reducing the “black box” nature and enhancing clinical interpretability.

In contrast to much of the literature, which depends on manual ROI annotation or strong slice-level labels [5,6,7], our study used a minimally supervised data preparation approach. Thanks to the MIL framework, a large number of unlabeled images could be effectively processed.

Additionally, patient-based data partitioning completely prevented data leakage. Many studies partition by image slices, leading to the same patient’s slices appearing in both training and testing sets, which artificially inflates performance. Our methodology avoided this flaw entirely.

The qualitative concordance observed between the Gated Attention MIL heatmaps and expert radiologist annotations suggests that the model focuses on clinically meaningful inflammatory patterns—particularly subchondral bone marrow edema—rather than on spurious image artifacts. This interpretability evidence, combined with the high statistical power of the independent test cohort (>0.99), further reinforces the credibility of the reported diagnostic performance despite the moderate sample size.

There are, however, several limitations. Although our study demonstrates robust performance, the dataset was sourced from a single institution. While significant efforts were made to ensure a high degree of patient diversity in terms of demographics and clinical presentations within our cohort, relying on a single center may inherently limit the model’s ultimate generalizability across different MRI scanners and varying institutional protocols. To establish the broad clinical reliability of the proposed Gated Attention MIL architecture, our future studies will prioritize multi-center validation using external datasets. Expanding the cohort with data from multiple institutions will be a critical next step to verify the model’s robustness in diverse real-world clinical settings. First, the study is based on data from a single center, necessitating validation of the model’s performance across different centers, scanners, and populations. The MRI systems used were predominantly 1.5 Tesla, and the model’s performance at varying field strengths was not assessed. Furthermore, only T2-weighted fat-suppressed sequences were evaluated; alternative sequences like STIR or post-contrast T1 were excluded, potentially limiting the model’s sensitivity to lesion diversity. The model was not validated on external datasets, leaving its generalizability unproven.

Future studies should incorporate multi-center data acquired using diverse scanners and imaging protocols to better evaluate generalization capacity. Hybrid CNN–Transformer architectures and advanced attention mechanisms could improve the precision of inflammatory lesion detection. Integrating MIL-generated attention maps with automatic lesion segmentation systems may enhance interpretability and ease integration into clinical decision support systems. Real-time deployment of such models could further promote the adoption of AI in clinical practice.

In conclusion, our Gated Attention MIL-based deep learning model demonstrated high diagnostic accuracy for detecting active inflammatory sacroiliitis on SIJ MRI. With accuracy (85.88%), sensitivity (92.86%), and specificity (79.07%), the model shows promising potential for both early diagnosis of axial spondyloarthritis and clinical application. Beyond classification, the MIL approach highlights clinically relevant slices, aiding decision-making.

Patient-based partitioning during training enabled realistic evaluation without data leakage. Despite being a single-center study with a broad patient population, the results suggest high generalizability. Nonetheless, broader validation through multi-center studies is warranted.

Ultimately, the proposed model offers an effective AI-based solution that can be integrated into clinical workflows for early and accurate sacroiliitis diagnosis. With further validation, its clinical utility can be significantly expanded.

## Figures and Tables

**Table 1:** Implementation and Training Configuration of the Proposed Gated Attention MIL Framework.

Parameter	Value
Backbone	ResNet-18 (ImageNet pre-trained)
Pre-training Strategy	All layers fine-tuned (no freezing applied)
Attention Dimensions	L = 512, D = 128, K = 1
Loss Function	Cross-Entropy Loss
Data Balancing	No explicit balancing; dataset approximately balanced (near 1:1 ratio)
Optimizer	Adam
Learning Rate	1 × 10^−4^
Weight Decay	1 × 10^−3^
Dropout Rate	0.5
Batch Size	4 (patient-level bags)
Epochs	20 (best validation F1-based model selection)
Inference Strategy	Test-Time Augmentation (Deterministic Horizontal Flip)

**Table 2:** MRI and Structural Findings in Patients with Active Osteitis (*n* = 276).

Finding	Patients with Active Osteitis (*n* =276)	Male (*n*, %)	Female (*n*, %)	Male Age (Mean)	Female Age (Mean)
Bone Marrow Edema	276 (100.0%)	104 (37.7%)	172 (62.3%)	35.0	39.6
Erosion	209 (75.8%)	83 (30.0%)	126 (45.7%)	35.6	40.8
Sclerosis	157 (56.9%)	62 (22.3%)	95 (34.4%)	35.2	41.0
Fatty Deposition	92 (33.3%)	47 (17.0%)	45 (16.3%)	37.9	41.8
Joint Space Narrowing	92 (33.3%)	49 (17.7%)	43 (15.6%)	36.0	38.3
Ankylosis	13 (4.6%)	11 (4.0%)	2 (0.7%)	36.1	58.5

Note: Data reflect proportions among 276 patients. Percentages are rounded; ages are sex-specific means.

**Table 3:** Clinical and Laboratory Findings in Patients with Active Osteitis (*n* = 276).

Finding	*n* (276)	Male (*n*, %)	Female (*n*, %)	Male Age (Mean)	Female Age (Mean)
Inflammatory Back Pain	224 (81.3%)	90 (32.7%)	134 (48.6%)	34.3	39.4
Morning Stiffness	198 (71.7%)	79 (28.6%)	119 (43.1%)	33.8	40.1
Psoriasis	13 (4.7%)	4 (1.4%)	9 (3.3%)	48.2	37.0
IBD	4 (1.4%)	3 (1.1%)	1 (0.3%)	29.0	46.0
Uveitis	8 (2.9%)	6 (2.2%)	2 (0.7%)	38.3	42.7
Arthritis	80 (29.0%)	33 (12.0%)	47 (17.0%)	36.1	44.7
Enthesitis	35 (12.7%)	11 (4.0%)	24 (8.7%)	36.5	43.7
Dactylitis	2 (0.7%)	1 (0.4%)	1 (0.4%)	30.0	44.0
Family History	30 (10.9%)	12 (4.3%)	18 (6.5%)	34.8	42.4
HLA-B27 Positivity	16 (5.8%)	10 (3.6%)	6 (2.2%)	31.0	37.7
CRP Positive	100 (36.2%)	43 (15.6%)	57 (20.7%)	33.9	42.1
NSAID Response	177 (64.1%)	52 (18.8%)	125 (45.3%)	35.9	40.3

Note: Each row represents a distinct clinical or laboratory finding. Numbers are scaled to a reference population of 276 patients. Sex-specific mean ages are reported for each category.

**Table 4:** Coexistence of Active Osteitis with Rheumatic Diseases (*n* = 276).

Finding	*n* (276)	Male (*n*, %)	Female (*n*, %)	Male Age (Mean)	Female Age (Mean)
Rheumatoid Arthritis	16 (5.8%)	5 (1.8%)	11 (3.6%)	20.6	35.6
Psoriatic Arthritis	5 (1.8%)	2 (0.7%)	3 (1.1%)	46.0	31.0
Behçet’s Disease	2 (0.7%)	2 (0.7%)	0 (0.0%)	32.5	-
Familial Mediterranean Fever	13 (4.7%)	8 (2.5%)	5 (1.8%)	29.0	36.6
Sjögren’s Syndrome	2 (0.7%)	0 (0.0%)	2 (0.7%)	-	59.5
Systemic Lupus Erythematosus	2 (0.7%)	0 (0.0%)	2 (0.7%)	-	40.5
Scleroderma	2 (0.7%)	0 (0.0%)	2 (0.7%)	-	40.0
Juvenile Rheumatoid Arthritis	2 (0.7%)	2 (0.7%)	0 (0.0%)	18	-

**Table 5:** Performance Metrics of the Model on the Test Set Using the TTA Strategy.

Metric	Baseline ResNet-18	Proposed System	Description
Accuracy	75.29%	85.88%	The proportion of correctly classified cases across all subjects
Sensitivity	85.71%	92.86%	The proportion of inflame patients correctly identified as positive
Specificity	65.12%	79.07%	The proportion of healthy individuals correctly identified as negative
F1-Score	0.7742	0.8667	The harmonic mean of sensitivity and positive predictive value (precision)

**Table 6:** Confusion Matrix of the Test Set (TP, TN, FP, FN).

Metric	ResNet-18	Proposed Method
True Positive (TP)	36	39
False Negative (FN)	6	3
True Negative (TN)	28	34
False Positive (FP)	15	9

**Table 7:** Comparison of Machine Learning and Deep Learning-Based Approaches for Sacroiliitis Detection.

Study	Method	Independent Test Accuracy (Acc)
**Nicolaes et al. (2025) [22]**	Deep Learning (731 patients)	74.00%
**Bressem et al. (2022) [18]**	Deep Learning (EULAR abstract)	75.00%
**Bressem et al. (2022) [20]**	Deep Learning (Radiology journal)	75–79%
**Faleiros et al. (2020) [6]**	Classical ML (MLP)	75–82%
**Liu et al. (2025) [21]**	Semi-supervised Radiomics	81.20%
**Roels et al. (2023) [23]**	Machine Learning (ResNet-18)	81.40%
**Zhang et al. (2024) [19]**	Radiomics + Clinical Hybrid Model	85.60%
**This Study**	**Gated Attention MIL**	**85.88%**

## Data Availability

The datasets generated or analyzed during the study are available from the corresponding author on reasonable request.
